# Cretaceous diversity of Schizaeales in Antarctica, *Escuderia livingstonensis* gen. et sp. nov., a permineralized fertile organ from Livingston Island, and its ecological implications combined with associated biota

**DOI:** 10.1093/aob/mcaf261

**Published:** 2025-10-22

**Authors:** Harufumi Nishida, Marcelo Leppe C, Aya Kubota, Julien Legrand

**Affiliations:** Chuo University, Tokyo 112-8551, Japan; Patagon Institute of Palaeobotany, Chiba 262-0023, Japan; Centro GEMA, Universidad Mayor, Santiago 8580000, Chile; Department of Geosciences, Osaka Metropolitan University, Osaka 536-8525, Japan; Faculty of Science, Shizuoka University, Shizuoka 422-8529, Japan

**Keywords:** Antarctica, Cretaceous, *Escuderia livingstonensis*, fern, *Ischyosporites*, permineralized, holophylogeny, Schizaeales, 3D reconstruction

## Abstract

**Background and Aims:**

Fossil records are indispensable for inferring the phylogeny of the leptosporangiate ferns. Permineralized fossils, which preserve anatomical details, can provide extensive anatomical information necessary for comparison with extant species. Fossil deposits of present-day Antarctica have proven particularly significant for elucidating the evolutionary history of divergence and diversification in the leptosporangiate ferns. In this study, we describe a newly discovered permineralized fossil of Schizaeales from the Cretaceous of Antarctica, with the aim of laying the groundwork for future phylogenetic analyses.

**Methods:**

The material studied is a permineralized fern reproductive organ preserved in a newly discovered silicified tuffaceous palaeosol from Livingston Island, Antarctica. The fossil-bearing horizon was inferred to correspond to the Williams Point Beds, dated to the mid-Cretaceous (Albian–Cenomanian). Serial sections were prepared using the peel technique, and a three-dimensional reconstruction of the entire organ was generated from the resulting image series using specialized software. In addition, high-resolution images of the remaining specimen were obtained using a newly developed method for ultrafine sequential surface imaging of fossils. Other biota components co-occurring with the target specimen in the matrix were studied using both peel sections and petrographic thin sections for anatomical and taxonomic studies.

**Key Results:**

The new fossil was designated as a new fossil taxon belonging to the order Schizaeales (family Schizaeaceae *s.l.*), and is described here as a new permineralized fern reproductive organ: *Escuderia livingstonensis* gen. et sp. nov. *In situ* spores were identified as a dispersed spore genus *Ischyosporites*, allowing the identification of one of the parent plants of that sporomorph. The new fern exhibits peculiar sympodial branching, which is unusual in both extant and fossil Schizaeales. The fossil-containing rock also preserved a gymnosperm ovulate organ of uncertain affinities, conifer shoots and woody root organs, and globose mycorrhizal nodules emerging from the conifer roots, representing part of the biota that coexisted with the fossil fern.

**Conclusions:**

The new genus *Escuderia* differs from both extant taxa and previously known fossil forms, exhibiting a primitive branching pattern and other anatomical features that provide new evidence for reconstructing the evolutionary history of the Schizaeales. The finding strengthens the hypothesis that Antarctica may have played a significant role in the diversification of the Schizaeales as well as of other leptosporangiate ferns. This study serves as a starting point for future comprehensive phylogenetic analyses of the Schizaeales, including undescribed permineralized fossils recently discovered in Japan and southern South America.

## INTRODUCTION

In the evolutionary history of leptosporangiate ferns, the role of Antarctica as a past centre of divergence and diversification for certain groups needs to be tested and is newly gaining attention. For the Cyatheaceae, Copeland (1947) proposed that their origin lies in Antarctica. More recently, a diverse array of fossilized cyatheaceous specimens, particularly permineralized fossils, have been reported from the Cretaceous of Antarctica and South Africa which was connected with Antarctica at that time as a part of Gondwana (Césari, 2006; Vera, 2009, 2013; Vera and Herbst, 2015; Vera and Césari, 2018; Silva *et al.*, 2025). Because permineralized fossils preserve not only external morphology but also rich anatomical details, they provide reliable evidence especially useful for phylogenetic analyses including both fossil and extant taxa. Molecular phylogenies of extant species can serve as a framework for constraining hypotheses about morphological character evolution in fossil taxa from which molecular data cannot be obtained. To infer a comprehensive phylogeny of leptosporangiate ferns including both extinct and extant taxa (holophylogeny, proposed by Nishida *et al.*, 2024), the discovery and description of as many permineralized fossils as possible is essential (Rothwell, 1999; Wikström *et al.*, 2002). However, as of this writing, fossil data to realize such future analyses appear to have accumulated primarily for the families Cyatheaceae and Osmundaceae. For Schizaeaceae *s.l.* (*sensu*Kramer, 1990; here equivalent to Schizaeales) general fossil data including impression/compressions have been extensively gathered, but only a limited number of fossils are used as calibration points even in the combined analysis by Wikström *et al.* (2002). Some permineralized fossils of Schizaeaceae have been reported from the Northern Hemisphere (Karafit and Stockey, 2008) but they remain insufficient to resolve relationships of fossil and living taxa in terms of morphological information and geographical distribution.

To date, more than 170 species of Schizaeaceae are widely distributed in tropical and subtropical regions mainly of the Southern Hemisphere (Kramer, 1990), although this number is not high compared to the sum of extant leptosporangiate ferns. The family is generally divided into four genera: *Lygodium* Sw., *Anemia* Sw., *Mohria* Sw. and *Schizaea* J.E. Smith (Kramer, 1990). Recent studies based on molecular phylogenetics reclassified the family into three separate families: Lygodiaceae, Schizaeaceae *s.s.* and Anemiaceae, as adopted in the Pteridophyte Phylogeny Group I (PPG I, 2016). However, in this study, the broader concept of Schizaeaceae is used, considering the possibility that phylogenetic relationships among these segregated families may shift once fossil taxa are included. Fossil records of the Schizaeaceae, primarily in the form of impressions/compressions, have been reported mainly from the Northern Hemisphere, and these fossils can be traced back to the Jurassic and possibly even to the Triassic (Karafit and Stockey, 2008; Taylor *et al.*, 2009). It is of note that fossil sporomorphs of Schizaeaceae/Schizaeales are character-rich and diagnostic, as represented by certain taxa such as *Cicactricosisporites* Potonié & Gelletich and *Ischyosporites* Balme.

Permineralized or charcoalified (i.e. anatomically preserved) fertile remains of Schizaeaceae have been reported from Japan (Stopes and Fujii, 1910; Yoshida *et al.*, 1996, 1997), the UK (Chandler, 1955), India (Bohra and Sharma, 1978; Sharma and Bohra, 1980) and North America (Hernandez-Castillo et al, 2006; Trivett *et al.*, 2006; Karafit and Stockey, 2008). Moreover, H.N. has so far collected six anatomically distinct permineralized fossils of the Schizaeaceae from the Cretaceous of Japan, southern Chile and Antarctica (Table 1). A preliminary comprehensive phylogenetic analysis of the Schizaeaceae, incorporating these specimens, has been carried out with acceptable results (Ohwi, 2023; Nishida *et al.*, 2024). While the present paper does not report the full results of the phylogenetic analysis, we provide a description of the new Antarctic material as a basis for future analyses.

**Table 1.** Schizaealean permineralized fertile organs at H.N.’s laboratory for future analysis (modified from Ohwi, 2023; Nishida *et al.*, 2024).

Specimen	Locality and horizon	Age	Tentative identification
AN120609 79C	Arida, Wakayama, Japan; Yuasa Formation	Barremian	*Stachypteris* Pomel
PWI-5P02	Livingston Island, Antarctica; Williams Point Beds	Albian–Cenomanian	Present specimen
150917G–27 1AC	Mikasa, Hokkaido, Japan; Mikasa Formation	Coniacian–Santonian	New species of *Schizaeopteris* Stopes and Fujii
COK61B2 (Sr1)	Cocholgue, Chile; Curanilahue Formation	Maastrichtian	New genus 1
CK5H1 (Sr2)	ibid	ibid	New genus 2

**Fig. 1.**
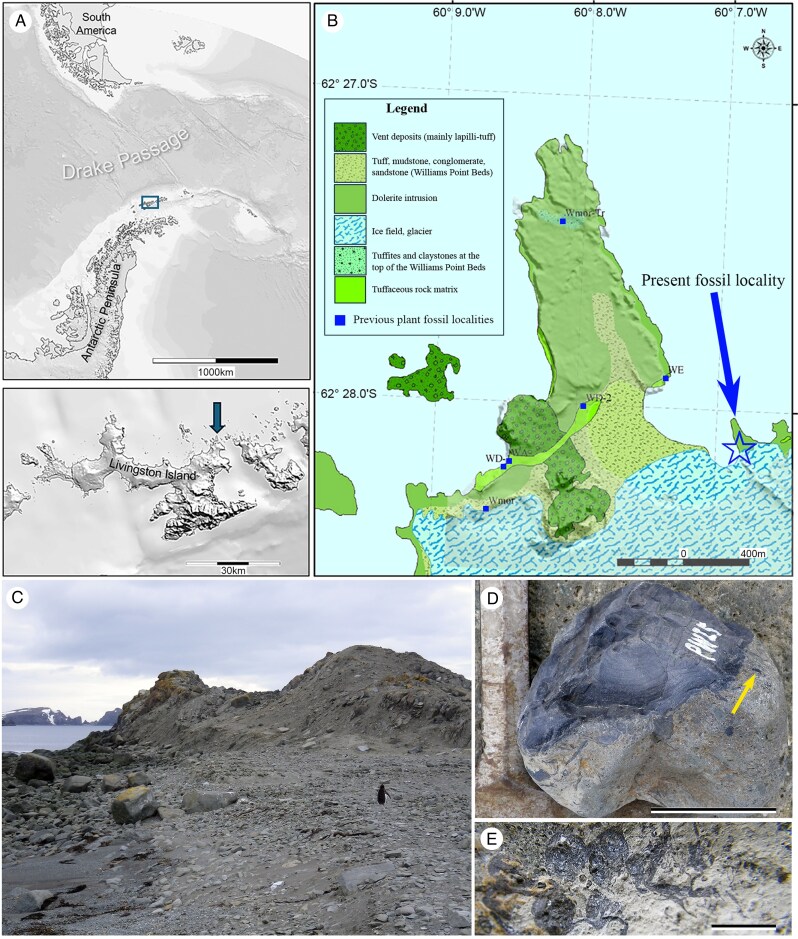
Fossil locality and studied material (PWI-5P02). (A) Location of Livingston Island and fossil site, Williams Point (arrow). (B) Fossil site (star) at Williams Point. (C) Exposed fossil site during the Antarctic summer. (D) Silicified fossil soil containing wood and plant fragments. The described specimen is exposed on the surface (arrow). Scale bar = 5 cm. (E) Fertile fern segment enlarged. Scale bar = 5 mm.

The material was found on the surface of a silicified palaeosol collected from Williams Point, Livingston Island, Antarctica (Fig. 1). Several fossil-containing rock samples were found isolated on exposed ground near Williams Point during field research by Instituto Antartico Chileno (INACH) in 2011. The material probably derived from the Williams Point Bed. The samples also contain a variety of permineralized plant organs such as leaf fragments, conifer wood, root systems, reproductive organs and possible soil microorganisms.

### Geological background

Williams Point, northeast of Livingston Island, is a 2-km area of exposed rock known for its abundance of xylological and foliar material. The strata at Williams Point are a sequence of conglomerates, sandstones and siltstones with interbedded tuffaceous horizons, accumulated in a fluvial environment (Chapman and Smellie, 1992). At Williams Point, a series of small hydroclastic sills and vents (ranging from 20 to 50 m in diameter) intrude a gently dipping and weakly lithified sedimentary sequence known as the Williams Point Beds (Hobbs, 1968; Smellie *et al.*, 1984; Georgiev *et al.*, 2023). Part of the palaeoflora described for Williams Point was initially assigned to the Triassic by Orlando (1967, 1968), an age determination later supported by several authors (Lacey and Lucas, 1981; Lemoigne, 1987; Barale *et al.*, 1994). However, subsequent collections at sites reported as Triassic (Rees and Smellie, 1989; Torres and Lemoigne, 1989; Chapman and Smellie, 1992) have revealed the presence of angiosperms, suggesting a probable Late Cretaceous age for the beds, also based on field and stratigraphic relationships (Rees and Smellie, 1989; Chapman and Smellie, 1992).

Based on palynological determinations, Chapman and Smellie (1992) assigned a minimum Campanian age to the Williams Point sequences, given the absence of *Nothofagidites* Erdtman ex Potonie, which rapidly expanded their distribution during the Campanian on the Antarctic Peninsula (Dettmann and Thomson, 1987; Askin, 1988; Dutra and Batten, 2000). More recent analyses of detrital zircons have yielded a maximum age of rock formation between the Cenomanian and Turonian, which constrains the fossiliferous rocks of the present report to a time span of approximately 83–93 Ma, in agreement with previous studies (Leppe *et al.*, 2007) based on biochrons of leaf impressions and palynomorphs. Recent K–Ar radiometric dating of these intrusive features has yielded ages of between 81 and 74 Ma, comparable to the Campanian (Georgiev *et al.*, 2023), indicating that the vents and conduits are younger than the surrounding fossiliferous rocks, corroborating an Albian–Cenomanian age for the floral assemblage (Rees and Smellie, 1989), which the present authors follow here.

## MATERIALS AND METHODS

The fossil material described in this study originates from a tuffaceous rock matrix recovered alongside blocks and boulders within the moraine located in the southeastern sector of Williams Point at the northeastern tip of Livingston Island (Fig. 1; 62°28.143′S, 60°06.834′W; Nishida *et al.*, 2016). This site is situated near locality P.1807, previously described by Cantrill (1997), to the east of Dragon Cove. The area is overlain by glacial deposits associated with the advance of the island’s ice cover (Fig. 1B, C).

The moraine clasts in the study area are predominantly of volcanic and pyroclastic origin. Many of the blocks consist of volcanic breccias containing centimetre-sized, angular, polymictic volcanic clasts, tuffaceous sands and silicified plant remains. Individual blocks can exceed 1 m in thickness. The tuff fragments include both vitreous and crystalline types, the latter sometimes featuring fragmented olivine crystals up to 2 cm in diameter, indicating mafic volcanic activity.

Preliminary observations reveal that some tuffaceous blocks contain silicified plant roots oriented perpendicular to the sedimentary bedding plane, bearing root hairs remarkably that are well preserved. This suggests the possible presence of *in situ* plants within palaeosols at the time of deposition.

The fern fossil was exposed on the surface of a silicified soil block (PWI-02) that also contains possible *in situ* (autochthonous) wood, roots and other transported plant remains of semi-*in-situ* (parautochthonous) nature deposited in the same matrix as the fern described here (Fig. 1D, E). The block was cut into smaller pieces by using a diamond slab-saw to obtain microscopic sections. Serial sections were prepared by the cellulose acetate peel technique using full-strength, commercial-grade (46 %) hydrofluoric acid (Joy *et al.*, 1956; Basinger and Rothwell, 1977). Peels were mounted on microscope slides with Canada Balsam. Petrographic thin sections were also made by Hayashi Thin Section Laboratory, Tokyo.

Optical microscope images were taken using a Pixera Pro 600ES camera on an Olympus BX50 microscope, a Leica Flexacam C1 camera on a Leica DM2500 differential interference contrast microscope, and a KEYENCE BZ-X710 all-in-one microscope. Images were processed using CombineZP for focus stacking and Photoshop 26.3.0 (Adobe, San Jose, CA, USA). Three-dimensional (3D) reconstruction of the fern was generated by using Amira 6.3 visualization software (Thermo Fischer Scientific, Waltham, MA, USA). Images of the rendering procedure and 3D movies are provided as supplementary information of this paper. All specimens and slides are deposited at Instituto Antartico Chileno (INACH), Punta Arenas, Chile.

**Fig. 2.**
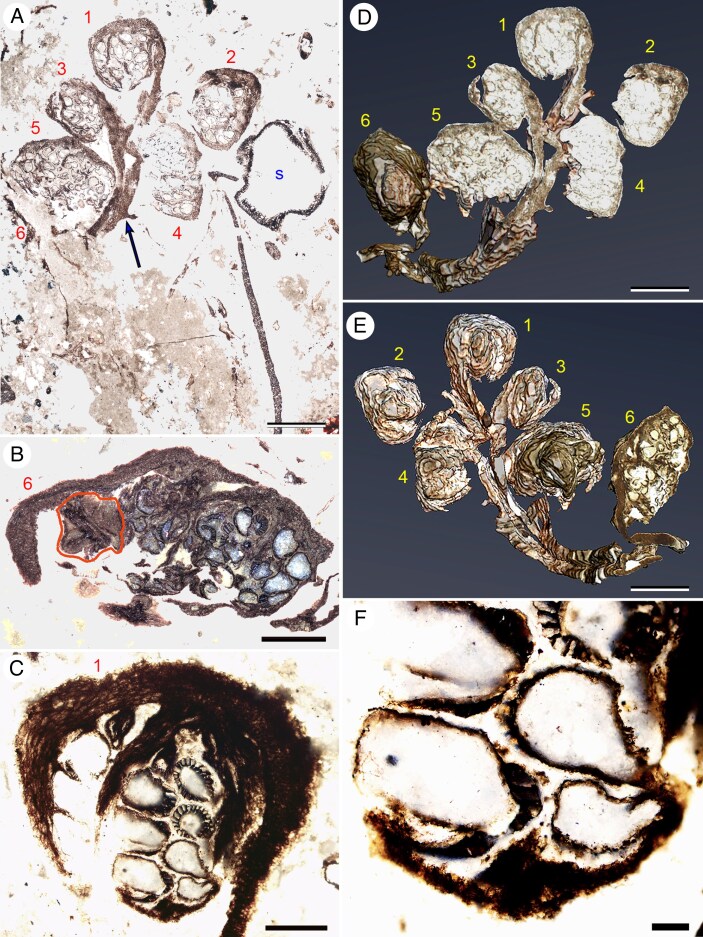
*Escuderia livingstonensis* gen. et sp. nov. (A) Peel section showing branching fertile segments and other organic fragments, including an unknown gymnosperm seed (S) terminating on an elongated stipe. Each of six fern segments is numbered. Arrow indicates base of the fern axis, which is enlarged in Fig. 3A. Scale bar = 1 mm. PWI-5P02-1top#0. (B) Lateral longitudinal section of unit 6 on polished surface next to PWI-5P02-1top#41 for high-resolution image analysis. Red contour shows conifer root with attached fungal nodules invading fern tissue. Scale bar = 1 mm. Image deposit number NMNS_DS00206_wl_01_0001.tif. (C) Median longitudinal section of unit 1, showing longitudinal rows of sporangia along ultimate segment. Scale bar = 0.5 mm. PWI-5P02-1top#5. (D, E) Three-dimensional rendering of the specimen. A rotating 3D image is provided as **Supplementary Data Video S1**. Numbers correspond to those in A. Scale bar = 1 mm. (F) Oblique section of ultimate segment abaxially bearing sporangia on each side of midrib. Top of sporangia seems directed toward midrib due to oblique section. Scale bar = 100 μm. PWI-5P02-1top#5.

Sequential cross-sectional images of one of the fertile units (#6) of the specimen (Fig. 2B) were obtained using a recently developed high-resolution grinding tomography technique (Paleobiology Laboratory, Hokkaido University, Sapporo, Japan; Ezaki *et al.*, 2023; Iba *et al.*, 2025). The rock sample containing the specimen was ground down using an automatic grinder, and each newly exposed surface was photographed at 4-µm intervals with a high-resolution digital camera (image resolution 19 008 × 12 672 pixels). The obtained image data are stored in the digital database of the National Museum of Nature and Science, Tsukuba, Japan, providing a serial number for each polished-surface image. Image reconstruction of the entire #6 unit has already been done and will be reported elsewhere.

## RESUTS

### Systematics

*Class*. Polypodiopsida Cronquist, Takht. & W. Zimm.

*Sub-class*. Polypodiidae Cronquist, Takht. & W. Zimm.

*Order*. Schizaeales Schimp.

*Family*. Schizaeaceae Kaulf. *s.l.* (*sensu*Kramer, 1990)

*Genus*. *Escuderia* H. Nishida, M. Leppe, A. Kubota et J. Legrand gen. nov.

*Generic diagnosis*. Leptosporangiate fern fertile organ consisting of axis branching sympodially more than six times; each branch terminating globose to head-shaped unit bearing sporangia. Each unit leafy, deeply pinnatifid, composed of one terminal and at least two pairs of lateral segments, and strongly enrolled abaxially; stomata abaxial and polocytic. Ultimate segment narrow, dorsiventral, oblong-ovate with broad base, rounded apex, and entire margin; venation 1-pinnate, free, bearing single, naked sporangium on abaxial side of each lateral vein, forming two rows of sporangia along each side of segment midvein; sporangium maturation on ultimate segment acropetal. Sporangium pyriform, slightly asymmetric, containing more than 64 spores, attached to lamina obliquely with thick and short basi-lateral base, and dehisces longitudinally; annulus terminal, single and complete, distally surrounding a multicellular terminal plate. Spores tetrahedral, globose, trilete, comparable to *Ischyosporites*.

*Type species*. *Escuderia livingstonensis* H. Nishida, M. Leppe, A. Kubota et J. Legrand sp. nov.

*Specific diagnosis*. Main axis ca. 0.5 mm thick, branching more than six times at >1.5–2-mm intervals. Fertile unexpanded unit round to oval, ca. 2.5 mm in diameter, with slender stipe 1.5–2 mm long, ca. 0.25 mm in diameter; expanding unit more than 5 mm long, consisting of one terminal and two to three pairs of lateral segments. Ultimate segment ca. 2–3 mm long, ca. 1 mm wide, margins slightly recurved abaxially, bearing total 8–10 sporangia in two rows along the midrib. Stomata ca. 12 µm long, 9 µm wide; stomium ca. 4 µm long, 2 µm wide. Sporangium pyriform 600–900 µm long, ca. 400 µm in diameter at thickest part, attached to lamina basi-laterally with thick multicellular base 4–5 cells wide, 5–6 cells high; annulus consisting of 17–26 thick-walled cells and pair of thin-walled marginal cells; terminal plate 100–150 µm in diameter, composed of ca. 15 or more thin-walled cells. Spores 33–38 μm in equatorial and 26–28 μm in polar diameter; laesurae straight, bounded by raised lips around 2.5 μm wide, and almost reaching the equator; contact area psilate, followed by coarse muri (1.5–3.5 μm wide) forming an irregular reticulum with lumina of various shapes and sizes (1.5–5 μm in diameter), generally interconnected near the equator and on strongly convex distal face.

*Holotype hic designatus.* Fertile segment in rock PWI-5P02. Deposition number CPAP 9720 (Colecciones Paleontológicas de Antártica y Patagonia) at Instituto Nacional de Antartica Chilena (INACH), slides PWI-5P02S-1top, series #1–#41. High-resolution images of serial sections after #41, obtained by using high-resolution grinding tomography technique are stored in database of National Museum of Nature and Science, Tsukuba, Japan, under deposition number NMNS_DS (Digital Specimen) 00206.

*Locality.* Williams Point, Livingston Island, Antarctica (Fig. 2A–C).

*Stratigraphic position and age.* Possibly Williams Point Beds, Albian–Cenomanian, mid-Cretaceous.

*Etymology. Escuderia* is dedicated to Julio Escudero Guzmán, a Chilean lawyer who participated in the drafting of the Antarctic Treaty of 1959 and lends his name to the Chilean Scientific Base ‘Escudero’ on King George Island. The specific epithet *livingstonensis* refers to the geographical origin of the specimen on the shore of Livingston Island.

### Description

The fossil is >1.6 cm long and ca. 0.7 cm wide and is composed of six globose or oval units (numbered 1–6 in Fig. 2A) each of which has one basal stipe concurrent alternatingly to a main axis ca. 0.5 mm thick. Distally from its base the main axis first dichotomizes to form two sub-axes adhering to each other for about 1 mm, then one axis departs sympodially to the lateral direction forming a stipe 1.5–2 mm long, ca. 0.25 mm in diameter and terminates in a globose or oval fertile unit (Fig. 2B, C). The remaining sub-axis represents the main axis that continues subsequent sympodial branching. The basal stipe of the structure #6 is not attached to the main axis, but its position suggests that it constitutes the basal-most unit of the same branching axis. The globose or oval unit is 2.2–3.8 mm in dimensions and composed of a circination of a pinnately lobed lamina (Fig. 2B, C). The 3D reconstruction of the entire structure reveals spatial branching patterns of the fossil (Fig. 2D, E; Supplementary Data Video S1). The most proximal unit 6 starts to expand and reaches more than 5 mm long (Fig. 2B).

**Fig. 3.**
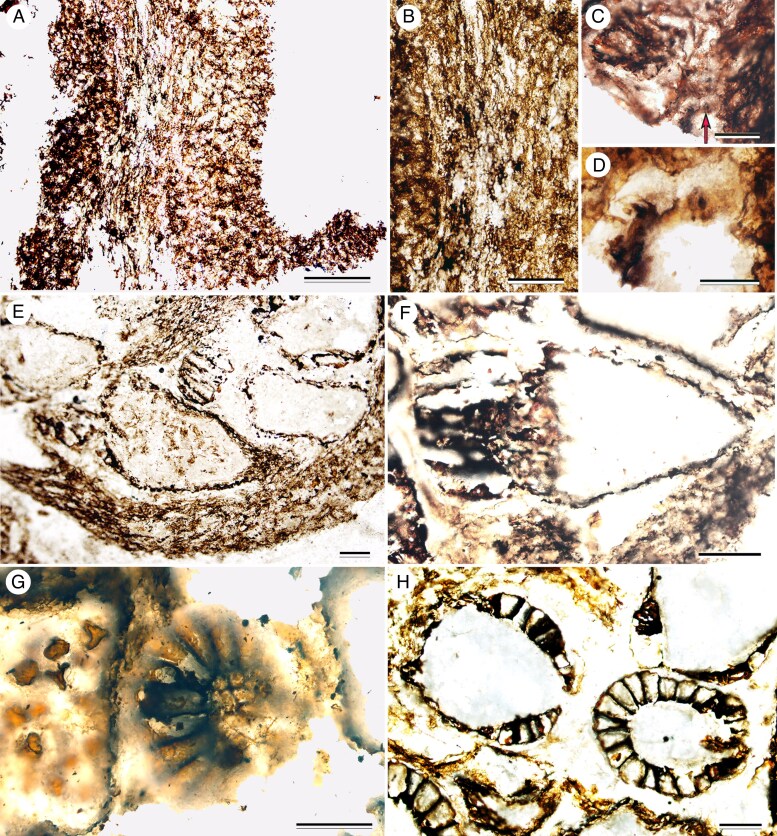
*Escuderia livingstonensis* gen. et sp. nov. (A) Longitudinal section of main axis at the portion shown by arrow in Fig. 2A. Tissues are poorly preserved. Scale bar = 0.1 mm. (B) Intensive fungal decay of tissues. Elongated cellular contours correspond to vascular tissue invaded by fungal hyphae. Scale bar = 100 μm. (C) Epidermal tissue preserving porocytic stomata (arrow). Scale bar = 100 μm. (D) Enlargement of C showing a well-preserved stomata to the right and another to the left intruded by fungal tissue. Scale bar = 20 μm. (E) Basal–lateral attachment of two sporangia with thick base to adaxial side of lamina. Scale bar = 100 μm. (F) Longitudinal section of a sporangium with terminal annulus. Scale bar = 100 μm. (G) Terminal plate cells on top of annulus. Some spores in other sporangium. Scale bar = 100 μm. (H) Cross-section of annuli of two sporangia, showing thick-walled annular cells and smaller marginal cells along the dehiscence suture. Scale bar = 100 μm. A, B, E, F: PWI-5P02-1top#0. C, D: PWI-5P02-1top#23. G: PWI-5P02-1top#3. H: PWI-5P02-1top#4.

The laminar ultimate segment is oblong-ovate with broad base, rounded apex and entire margin, ca. 2–3 mm long and ca. 1 mm wide, slightly incurved abaxially, and bears in total 8–10 sporangia arranged in two rows along each side of the midrib (Fig. 2B–F). No indusium-like covering of the sporangium was observed. Sporangium size diminishes toward the distal direction of each ultimate segment, evidencing acropetal maturation of sporangia. The venation of the ultimate segment is 1-pinnate and free judging from the sporangial position, but a detailed pattern was not traceable owing to poor preservation of the vascular as well as the fundamental tissues caused by organismal decay mainly due to fungal intrusion (Figs 2C, F and 3A, B). One paradermal section of poorly preserved abaxial epidermis shows two stomata that occur at the internal periphery of the epidermal cell (Fig. 3C, D), which is comparable to the porocytic morphology typified by van Cotthem (1970). Stomata are ca. 12 µm long and 9 µm wide with a stomium ca. 4 µm long and 2 µm wide. One stoma has been destroyed by fungal tissue invasion through the stomium (Fig. 3D).

**Fig. 4.**
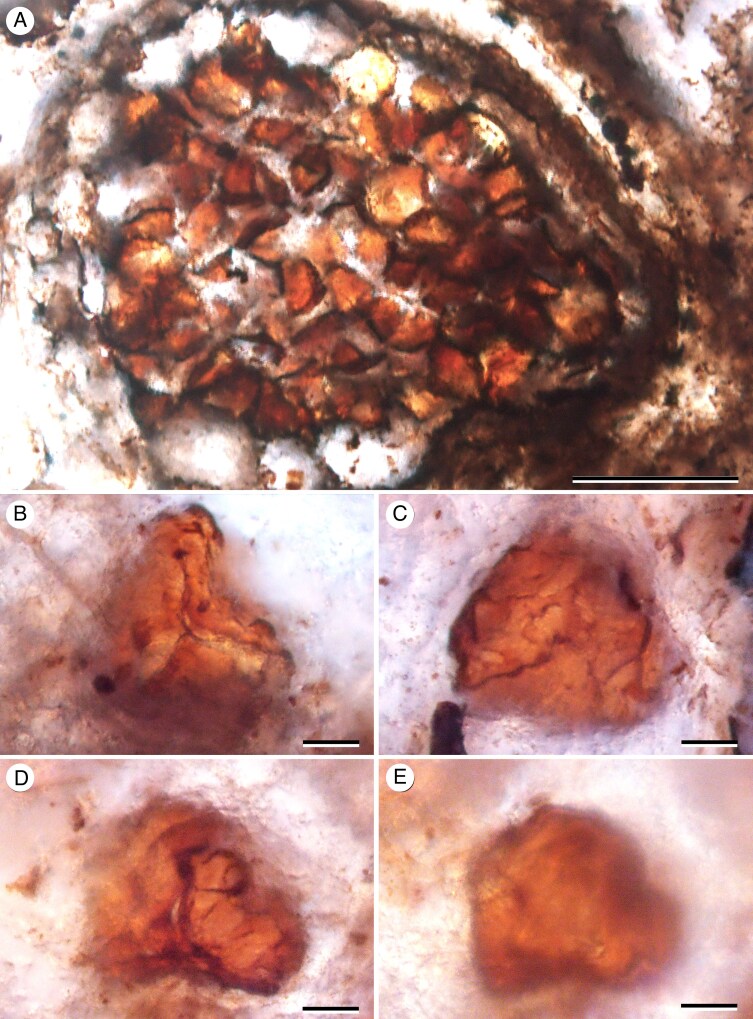
*In situ* spores of *Escuderia livingstonensis* gen. et sp. nov. (A) Sporangium containing >64 immature spores of *Ischyosporites* cf. *volkheimeri*. Scale bar = 100 μm. PWI-5P02-1top#3. (B–E) *Ischyosporites* cf. *volkheimeri* observed under differential interference contrast microscopy. Scale bars = 10 μm. (B) Proximal face. PWI-5P02-1top#1. (C) Distal face showing incomplete reticulum. PWI-5P02-1top#1. (D, E) Proximal (D) and distal (E) faces of the same spore. PWI-5P02-1top#3.

Sporangium is pyriform, slightly asymmetrical, 600–900 µm long, ca. 400 µm in diameter at the thickest part, and attached to the abaxial side of the ultimate segment basi-laterally with a thick multicellular base 4–5 cells wide, 5–6 cells high (Fig. 3E, F). The sporangial annulus is terminal and complete, consisting of 17–26 thick-walled cells and a pair of thin-walled marginal cells along the dehiscent slit (Fig. 3E–H). At the top of the sporangium is a terminal plate 100–150 µm in diameter, composed of thin-walled cells, ca. 15–19 in number estimated from partially preserved plate cells (Fig. 3G). The sporangium contains more than 64 tetrahedral spores (Figs 3E, G and 4A). A certain number of spores are dispersed from the sporangium and remain in-between the fern tissue. Spore walls are not well-preserved probably because they are at an early maturation stage and some have suffered fungal attack. The identification and taxonomic treatment of the spores are separately described below.

### Spores *in situ*

Genus *Ischyosporites*Balme, 1957

Type species: *Ischyosporites crateris*Balme, 1957

*Ischyosporites* cf. *volkheimeri*Filatoff, 1975

#### Description

Trilete microspore; amb triangular with sides straight or slightly convex and apices rounded (Fig. 3B–E). The laesurae are straight, bounded by raised lips around 2.5 μm wide, and almost reach the equator. Contact area psilate, followed by coarse muri (1.5–3.5 μm wide) forming an irregular reticulum with lumina of various shapes and sizes (1.5–5 μm in diameter), generally interconnected, near the equator and on the distal face. Distal face strongly convex. Equatorial diameter 33–38 μm; polar diameter 26–28 μm. Exine about 1 μm thick.

#### Botanical affinities

Lygodiaceae–Schizaeaceae (Balme, 1957, 1995; van Konijnenburg-van Cittert, 1981, 1991; Tryon and Lugardon, 1990; Dettmann, 1994). *Ischyosporites* has been reported *in situ* in the Jurassic fossil plants *Klukia exilis* (Phil.) Raciborski, *K. westii* (Phil.) Raciborski and *Stachypteris spicans* Pomel (see Balme, 1995 for details).

#### Distribution

Wide distribution in Jurassic to Tertiary sediments. In Antarctica, the genus is reported from the Middle Jurassic (Bajocian) to Palaeogene (Eocene) (e.g. Dettmann and Thomson, 1987; Dolding, 1992; Keating *et al.*, 1992; Riding *et al.*, 1998; Barreda *et al.*, 1999; Truswell *et al.*, 1999; Contreras *et al.*, 2013; di Pasquo and Martin, 2013; Amenábar *et al.*, 2014; Bomfleur *et al.*, 2014; Chen *et al.*, 2015; Thompson *et al.*, 2022). In southern South America, which had a geographical connection with Antarctica during the Cretaceous, *Ischyosporites* is also reported, for example, from the Aptian of Santa Cruz (Perez Loinaze and Llorens, 2018) and Albian of La Rioja, Argentina (Mego *et al.*, 2019).

### Associated biota

**Fig. 5.**
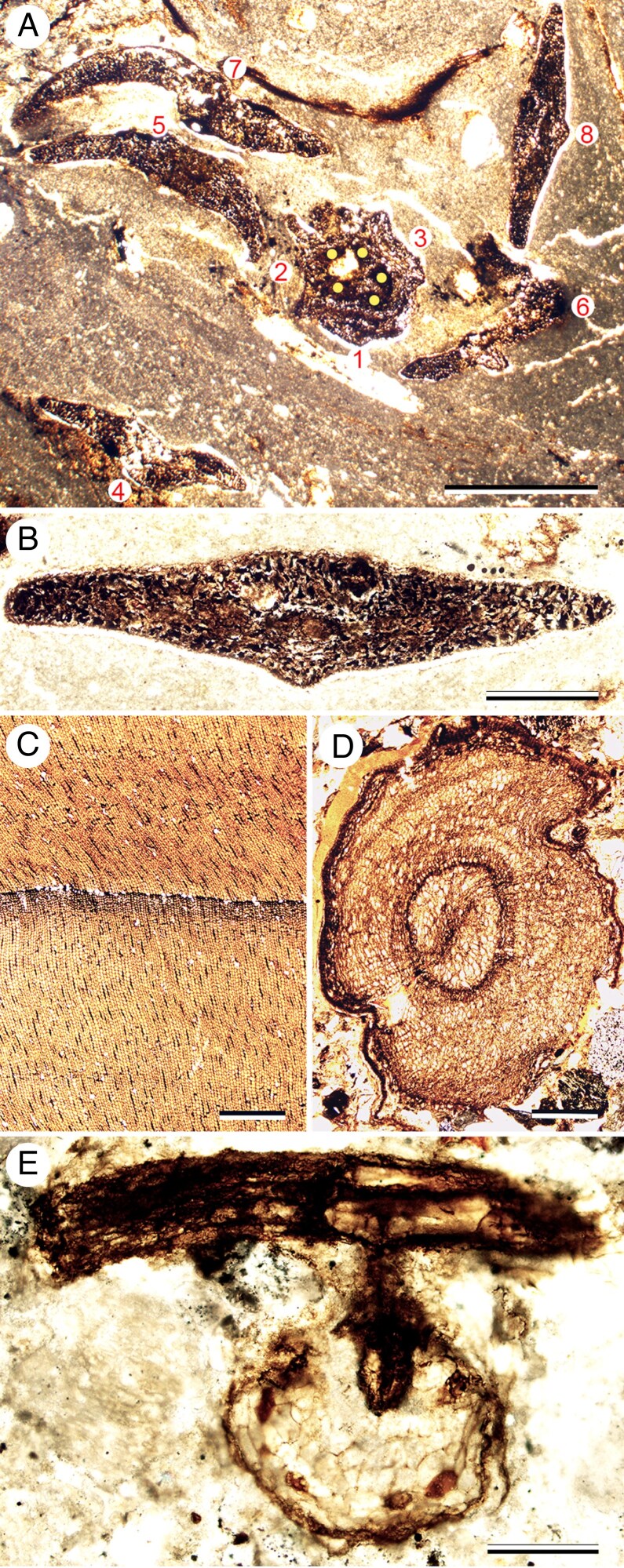
Plants and fungi associated with *Escuderia livingstonensis* gen. et sp. nov. in the same rock sample, PWI-5P02 (Fig. 2D). (A–D) Petrographic thin sections. (A) Conifer shoot bearing flat leaves (numbers) in 2/5 phyllotaxy. Shoot axis has five vascular strands (yellow circles). Scale bar = 1 mm. PWI-5P02①#1. (B) Leaf 8 in A. Note single vascular bundle in the midrib. Upper side adaxial. Scale bar = 0.3 mm. (C) Cross-section of thick wood in Fig. 2D, showing a seasonal ring marked by narrow late wood. White holes in wood are the product of fungal decay. Scale bar = 0.5 mm. PWI-5P02①wood. (D) Cross-section of a woody root derived from thicker trunk in D, showing primary and secondary wood, and periderm. Note ultimate root trace in the secondary wood. Scale bar = 0.5 mm. PWI-5P02①wood. (E) Arbuscular mycorrhizal nodule attached to a conifer root. Note vascular bundle emerging from parental root. Scale bar = 200 μm. Peel WI-5P02-1top#23.

Other plant remains are also preserved in sample PWI-5P02 (Figs 1D and 5). Although their histological preservation is not good owing to intensive fungal attack, some are identifiable to different taxonomic levels and provide floristic and ecological perspectives of the original plant assemblage.

One noteworthy structure is a seed-plant reproductive organ associated with *Escuderia* (Fig. 2A). It consists of a round, naked ovule ca. 4 mm in diameter borne on a short stipe ca. 1 mm long and 0.1–0.4 mm in diameter. The stipe bifurcates nearly horizontally at its top, leaving a flat tip and a slightly raised shoulder to the other side of the ovule, which possibly represents the base of another detached ovule. The ovule wall or integument is thin, at least two-layered and without recognizable vascular bundles due to tissue decay. Judging from the wall thickness and tissue composition, though incomplete, the ovule is unitegmic. In serial sections the ovule elongates longitudinally to exhibit an oval shape and the micropyle opens at the distal end. The structure therefore represents a unitegmic gymnospermous ovule without any additional external envelope. This is not discussed in detail below.

Petrographic thin sections of the parent rock reveal conifer leafy shoots and isolated leaves similar in anatomical features to each other (Fig. 5A, B). The shoot axis is 0.7–0.8 mm in diameter with five vascular bundles and bears flat and elongated leaves in spiral 2/5 phyllotaxy (Fig. 5A). Associated leaves are falcate and flat or slightly recurved adaxially in cross-section, ca. 0.5 mm wide at the base attached to the main axis, ca. 1 mm or less at the distal widest portion, and more than 4 mm long estimated from one longitudinally sectioned incomplete fragment in the same matrix. The leaf tissues are highly damaged but the presence of a single vascular bundle in the midrib and almost isomorphous mesophyll tissues are confirmed (Fig. 5B).

The largest components in the rock are wood fragments of various sizes. Petrographic thin sections revealed gymnosperm wood features with marked seasonal rings, although cellular details of the wood tissues have been destroyed by fungal intrusion, making further taxonomic identification difficult (Fig. 5C). The thickest wood has more than six seasonal rings counted from the preserved periderm (only one ring boundary is shown in Fig. 5C). Abundant white dots that randomly occur in the secondary wood are the result of fungal decay. A smaller woody axis possibly derived from the thickest wood shows the entire cross-section and has central primary xylem surrounded by secondary wood and outermost periderm (Fig. 5D). The primary wood exhibits a diarch structure and an ultimate root trace is found embedded in the secondary wood, suggesting its endogenous origin. These woody axes possibly represent an underground root system of trees that grew *in situ.* The ultimate roots are slender, ca. 0.2 mm in diameter, frequently found in the silicified soil and often penetrate other plant fragments including *Escuderia* (Fig. 2B, red contour).

Some ultimate roots bear spherical nodular structures, ca. 0.5 mm in diameter, each emerging at a right angle from the mother root surface (Figs 2B and 5E). The structure is connected to the root with a columnar vascular tissue surrounded by thick parenchymatous cortex, and resembles mutualistic arbuscular mycorrhizal nodules reported from roots of extant and fossil conifers (Nunes *et al.*, 2020). The soil matrix further contains remains of permineralized microorganisms.

## DISCUSSION

### Placement in Schizaeales and comparison with fossil taxa

**Fig. 6.**
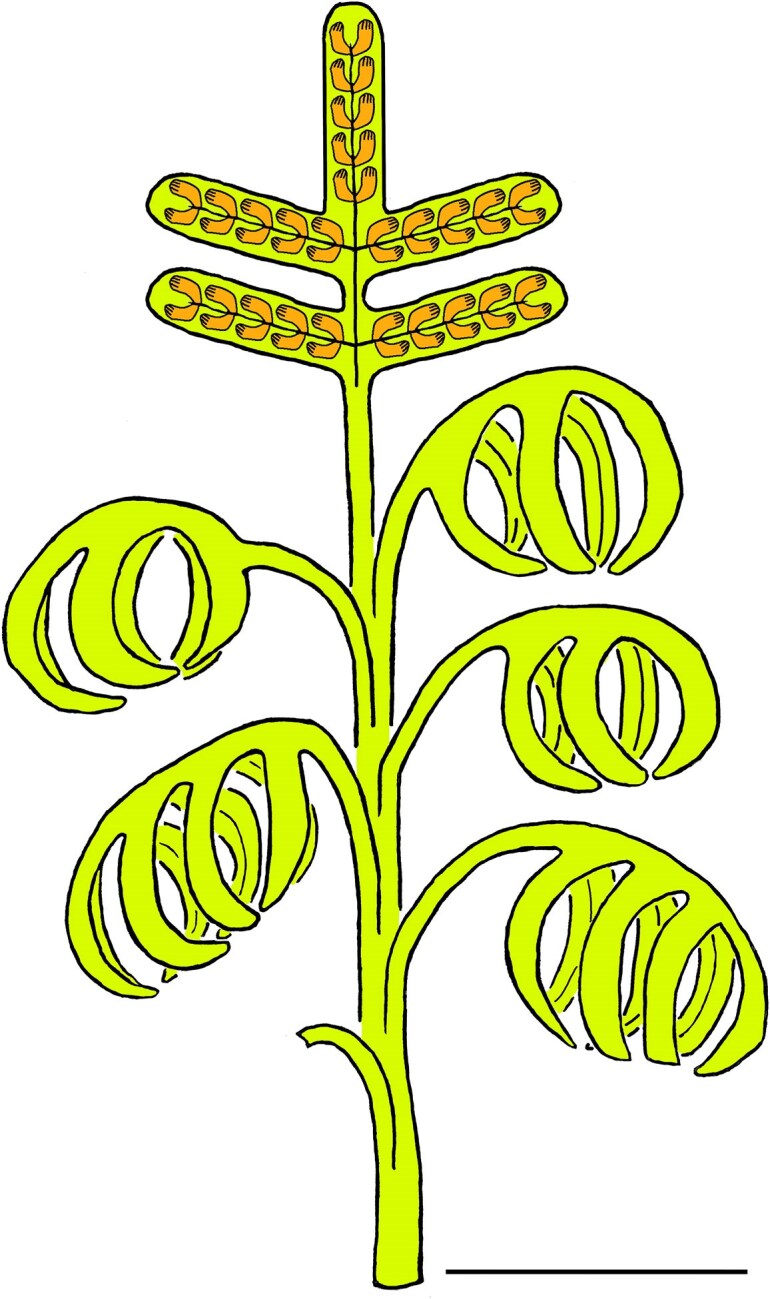
Suggested reconstruction of *Escuderia livingstonensis* gen. et sp. nov. Ultimate unit is illustrated open showing sporangia positions. Sporangia of circinate fertile units are omitted. Scale bar = 5 mm.

The simple and complete terminal annulus of the sporangium confirms affinity of the fossil to the family Schizaeaceae *s.l.* of Schizaeales (Kramer, 1990). Double sporangial rows on the abaxial side of a narrow terminal fertile segment or axis are typical of the family, except *Mohria*. Based on the 3D reconstruction (Fig. 2D, E) and visual observations of serial images obtained from the new high-resolution grinding tomography technique (Fig. 2B), a reconstruction of *Escuderia* is suggested (Fig. 6). The habit and possible dimorphy of the frond, which occurs to varying degrees in the family, remain unknown.

The most peculiar and unique feature of *Escuderia* is the ramification pattern of the non-laminar main axis, which is sympodial starting from a basal dichotomy of the main axis. The pinnatifid laminar unit on top of each lateral branch of *Escuderia* resembles fertile penultimate segments of *Anemia*, though the latter constitute larger compound pinnae unlike the lamina-less proximal axis of *Escuderia* (Labiak *et al.*, 2015). *Anemia* also differs from *Escuderia* in its strongly sculptured cicatoricose spores (Tryon and Lugardon, 1990). The polocytic stomata of *Escuderia* occur also in *Anemia* and *Mohria* (Kramer, 1990) but this is not a character that strengthens their taxonomic affinity by itself. Strongly reduced fertile penultimate pinnules consisting of a cluster of elongated ultimate segments occur in some species of *Lygodium*, such as the North American species *L. palmatum* (Bernh.) Sw. and the Eocene impression fossil *L. skotsbergii* Halle from south Chile (Andrews and Boureau, 1970). These segments are palmately arranged unlike in *Escuderia*, and the genus *Lygodium* differs from the former in the presence of indusium-like sporangial protection (Kramer, 1990). Moreover, *Escuderia* differs from any of the extant four genera (*sensu*Kramer, 1990) in its semi-basal sporangial attachment to the lamina in contrast to the lateral attachment in *Lygodium*, and basal attachment in others. The *Ischyosporites*-type spores do not occur in extant schizaealeans (Tryon and Lugardon, 1990).

As far as we know, schizaealean fossils comparable to the entire morphology of *Escuderia* have not been reported. Some fossils of both impression/compressions and permineralizations as old as the Jurassic or even Triassic resemble *Escuderia* in having abaxially enrolled fertile pinnules with two abaxial rows of sporangia (summarized in Karafit and Stockey, 2008). Among them, anatomically comparable permineralized forms are: *Anemia poolensis* Chandler (Palaeocene/Eocene, England: Chandler, 1955, reviewed in Collinson, 2001); *Anemia quatsinoensis* Hernandez-Castillo, Stockey & Rothwell (Valanginian/Hauterivian, Canada); *Schizaeopteris* Stopes & Fujii (Turonian–Coniacian, Japan: Stopes and Fujii, 1910; Yoshida *et al.*, 1996); and *Paralygodium* Yoshida, H. Nishida & Nishida (Coniacian–Eocene, Japan and Canada: Yoshida *et al.*, 1997; Trivett *et al.*, 2006; Karafit and Stockey, 2008). *Anemia poolensis*, which is partly pyritized and has poorly preserved histology, differs from *Escuderia* in external architecture and smooth spores. *Anemia quatsinoensis* represents the oldest species of the genus based on fertile as well as vegetative external morphology and anatomy; its cicatricose spores are distinct from those of *Escuderia*. *Schizaeopteris* is anatomically comparable to *Anemia* and distinguished from *Escuderia* in leaf architecture, basal/basi-lateral sporangium attachment and also cicatricose spore sculpture. *Paralygodium* is characterized by its palmate pinnules and smooth to psilate spores, some comparable to the dispersed spore genus *Deltoidospora* Miner as in *P. meckertii* Karafit & Stockey (Karafit and Stockey, 2008). Altogether, based on its characteristic external morphology and anatomical features, we thus assign the new Antarctic fossil to a new permineralized fossil genus *Escuderia*.

Basi-lateral sporangia are reported in some fossil taxa according to the preliminary character distribution matrix of both extant and fossil taxa of the Schizaeales by Ohwi (2023): they are *Anemia poolensis*, *Paralygodium*, *Schizaeopteris* (basal/basi-lateral) and *Lygodium bierhorstiana* Gandolfo, Nixon, Crepet & Ratcliffe from the Turonian of the USA (Gandolfo *et al.*, 2000). This character may represent a plesiomorphic feature found in the stem groups of Schizaeales. Some Jurassic to Early Cretaceous taxa, such as *Klukia* Raciborski and *Stachypteris* Pomel (Harris, 1961; van Konijnenburg-van Cittert, 1991), and *Klukiopsis*Deng and Wang (2000), are regarded as the earliest members of the Schizaeales (Wikström *et al.*, 2002; Taylor *et al.*, 2009). Their sporangial attachment has not yet been clarified because of the lack of anatomically preserved material. Barremian permineralized segments preliminarily identified as *Stachypteris* have been recovered from Wakayama Prefecture, Japan (Table 1). However, as no microscopic sections preserving the sporangial base have yet been identified, further investigation is planned.

Spore number per sporangium is generally large in primitive groups of ferns (Bower, 1923). In Schizaeales, sporangia typically contain numbers greater than 128. Among permineralized fossils comparable to *Escuderia livingstonensis*, 128–256 per sporangium is estimated in *Paralygodium meckertii* based on 105–159 counts (Karafit and Stockey, 2008). Considering the close sporangium and spore sizes of *Escuderia* and *P. meckertii* (600–690 µm long, ca. 300 µm wide and 33–38 μm in equatorial diameter vs. 360–468, 216–300 and 33–42 μm, respectively), similar spore numbers can be estimated for *E. livingstonensis* assuming comparable spore size and shape.

### *In situ* spore identification and remarks

In the Cretaceous dispersed spore assemblages of Antarctica, *Ischyosporites* is represented by *I. crateris* Balme, *I. punctatus* Cookson and Dettmann, and *I. volkheimeri* Filatoff. *Ischyosporites crateris* is different from our specimen in the subcircular to circular lumina shape of its reticulum, and from *I. punctatus* in its distinctive pitted muri. *Ischyosporites volkheimeri* corresponds in size, shape and characteristics of its sculpture with our specimen, although the immature state of the spores observed prevents definite identification. We therefore identify the *in situ* spores of *Escuderia livingstonensis* as *I.* cf. *volkheimeri*.

*Ischyosporites volkheimeri* is reported in Antarctica from the Bajocian–Oxfordian of the Mac Robertson shelf (Truswell *et al.*, 1999) and the Aptian–lower Maastrichtian of James Ross Island (Dettmann and Thomson, 1987; Keating *et al.*, 1992; Riding *et al.*, 1998; Barreda *et al.*, 1999; di Pasquo and Martin, 2013). It is noteworthy that *Ischyosporites* is reported from *Klukia exilis*, *K. tyganensis* Krassilov and *Stachypteris spicans* Pomel, which represent the earliest members of the Schizaeales in the Jurassic. In the sporangia of a Barremian fertile segment from Wakayama, Japan, one of the authors (J.L.) preliminarily identified *in situ Ischyosporites* spores as *Stachypteris*.

#### Diversity of Schizaeales in Antarctica and future holophylogenetic analysis

A schizaeoid fertile organ containing spores comparable to *Cicatricosisporites*, which is generally attributed to anemioid schizaleans, is also found in a different rock sample from the same locality (PWI-5P01B), though we do not intend to describe it here. Chapman and Smellie (1992) mentioned the scarce presence of *Cicatricosisporites* from the Williams Point Beds without showing images. From the Campanian of James Ross Island, a pinnate segment resembling the form genus *Schizaeopsis* Berry, which is similar to extant *Schizaea dichotoma* (L.) Sm., has been described (Iglesias, 2016). These findings indicate the coexistence of possible stem groups related to several monophyletic lineages within extant Schizaeales (Wikström *et al.*, 2002) as well as the separate ancestral group represented by *Escuderia* in the Cretaceous Antarctic flora. *Escuderia* exhibits a combination of probable primitive characters: (1) sympodial branching originated from a basal dichotomy, (2) basi-lateral attachment of sporangium and (3) *Ischyosporites* spores shared by some earlier schizaealeans.

Impression/compression fossils of the Schizaeales seem to be less common in Antarctica than those of other taxonomic groups such as Osmundales, Matoniales, Gleicheniales and Cyatheales. This might be due partly to the smaller habit of schizalean ferns, which could minimize preservation probabilities. However, clearly Schizaeales formed an important component of the Cretaceous Antarctic flora as well as of Gondwana since the Jurassic (McLoughlin, 2001). Permineralized material such as *Escuderia* in combination with fossils of other preservation types provides reliable morphological information that is needed for future holophylogenetic analysis (Table 1).

#### Biota and environment

Numerous plant fossils have been reported from the Williams Point Beds owing to seasonal retreat of the ice sheet during the summer months (Orlando, 1967, 1968; Lacey and Lucas, 1981; Banerji and Lemoigne, 1987; Rees and Smellie, 1989; Torres and Lemoigne, 1989; Chapman and Smellie, 1992; Philippe *et al.*, 1993; Cantrill, 1997; Poole and Cantrill, 2001). These fossils represent a diverse array of taxa preserved as impressions of leaves, permineralized wood and rhizomes, and palynomorphs. Although a detailed correlation of the fossil-bearing horizons is not attempted here, a comprehensive view of the composition of the Williams Point Beds flora can be drawn. Although available data are insufficient for a detailed comparison of taxonomic diversity, the relative amount of spore and pollen production follows the order of pteridophytes, gymnosperms and angiosperms (Chapman and Smellie, 1992).

Chapman and Smellie (1992) reported fern spores occupying at most 45 % of the palynoflora from the lower member of the Williams Point Beds. About 13 species of fern spores have been identified without showing composition details. Only a few sporomorphs, *Osmundacidites*?, *Cyathidites*?, *Cyatheacidites*? and one trilete sporomorph are figured as scanning electron micrographs, none of which was comparable to *Ischyosporites* or other schizaeoid spores. From a locality very close to the present fossil site, a permineralized Osmundaceae rhizome, *Ashicaulis livingstonensis* Cantrill, was described (Cantrill, 1997). The rhizome was recovered as a single specimen from a tuffaceous block but it is not clear whether it is parautochthonous like the current material; therefore, despite their spatial proximity, the fossil horizons are probably different. *Escuderia* adds a new schizaeoid fern to the Williams Point Beds flora.

The silicified palaeosol containing *Escuderia* includes either autochthonous or parautochthonous plant material and provides new evidence to unveil the floristic composition. In association with *Escuderia*, we observed a gymnosperm reproductive organ, conifer shoots, leaves, root wood and rootlets bearing mycorrhizal root nodules (Figs 2A and 5).

The affinity of the ovule-bearing organ (Fig. 2A) remains uncertain. The naked unitegmic ovule terminated on possibly the bifurcated end of an elongated stipe is comparable to the female organ of the Ginkgoales, which had a worldwide distribution including Antarctica during the Cretaceous (Crane, 2013). However, further histological details are needed to confirm the attribution.

The conifer shoots and leaves (Fig. 5A, B) are comparable to those of either Araucariaceae or Podocarpaceae based on the morphology, anatomy and floristic composition of Cretaceous Antarctica (Francis *et al.*, 2007). A slender flat leaf with a single vascular bundle in the midrib and spiral phyllotaxy occurs in some species of the Araucariaceae and Podocarpaceae (Page, 1990). In the Podocarpaceae, anatomical features such as the distribution of resin canals and transfusion tissues are crucial for generic identification (Knopf *et al.*, 2012). However, those features are not preserved in our fossil leaf. The present shoots and leaves resemble the podocarpalean impression genera *Bellingshausium* Cantrill or *Pagiophyllum* Heer, the latter a form genus of varying affinity including the Araucariaceae and Podocarpaceae, both from the Albian of James Ross Island (Cantrill and Falcon-Lang, 2001). *Pagiophyllum* has also been reported from Williams Point (Banerji and Lemoigne, 1987).

The woody conifer root is attributable to either Araucariaceae or Podocarpaceae, although further identification is difficult due to insufficient preservation (Figs 1D and 5C, D). According to Poole and Cantrill (2001), the conifer wood flora of the Williams Point Beds comprises two species of Araucariaceae and three taxa of Podocarpaceae. Some wood pieces preserved in other silicified soil samples from our fossil locality exhibited araucarian tracheary pitting, though further comparison is needed to confirm the identity of the new wood materials.

Ultimate roots bearing mycorrhizal nodules are found in the woody root in the same sediment (Fig. 5D, E). Hyphal arbuscules characteristic of arbuscular mycorrhizal nodules (AMNs) are confirmed inside the cortical cells of the nodules, though anatomical details are not described here. Mesozoic fossil records of coniferophyte AMNs are scarce, of which three are permineralized and two are from the Southern Hemisphere (Moisan *et al.*, 2021). The present nodules are most similar in anatomy to the AMNs colonizing roots of Araucariales (Araucariaceae and Podocarpaceae) from the Jurassic of Argentinian Patagonia (Nunes *et al.*, 2020). A different form of permineralized AMNs, borne on the roots of the Voltzialean conifer *Notophytum krauselii* Meyer-Berthaud & Taylor, was reported from the Triassic Fremouw Formation of the Central Transantarctic Mountains, Antarctica (Schwendemann *et al.*, 2011; Harper *et al.*, 2015). The impressions of AMNs described by Cantrill and Falcon-Lang (2001) from the Albian Neptune Glacier Formation of Alexander Island, Antarctica, are similar to our fossils in form and size. The host tree is presumed to belong to the Podocarpaceae based on the taxonomic composition of coexisting fossils. The new AMNs indicate that the symbiotic relationship between mycorrhizal fungi and conifers persisted in Antarctica from the Triassic through the Cretaceous. In particular, the form of symbiosis observed in present-day Araucariaceae/Podocarpaceae probably continued from those found in southern South America and Antarctica since the Jurassic.

Angiosperms are an important floristic element in Antarctica given their worldwide rise throughout the Cretaceous. The mid-Cretaceous Williams Point Beds preserve mega- and microfossils that evidence the early stage of Antarctic angiosperm diversification (Chapman and Smellie, 1992). Our new material is expected to be a source of structurally preserved new angiosperm remains.

A rich record of fossil leaves, wood, flowers and pollen from the Antarctic Peninsula and the Transantarctic Mountains are reviewed by Francis *et al.* (2007) in terms of climatic and biota change from the Cretaceous greenhouse to the Neogene ice world. The Williams Point Beds flora represents a green utopia during the warm period of Antarctica (e.g. Crame, 1992). The new permineralized soils that contain a diverse array of organisms can contribute to reveal part of the complex ecosystems in Cretaceous Antarctica.

## CONCLUSIONS

The mid-Cretaceous (Albian–Cenomanian) fern *Escuderia livingstonensis* gen. et sp. nov. is described as a basal member of the family Schizaeaceae *s.l.* (Schizaeales). *Escuderia* represents the first anatomically preserved Schizaeaceae fossil discovered in Antarctica. It is reconstructed three-dimensionally using ordinary image rendering and a new high-resolution tomography technique (Ezaki *et al.*, 2023; Iba *et al.*, 2025), opening new possibilities for future research methodologies on permineralized plant fossils. The spores are identified as the sporae dispersae genus *Ischyosporites*, linking this spore morphogenus to one of its parent plants. The overall reconstructed morphology of *Escuderia*, along with the anatomical features of the sporangia and spores, exhibits primitive characteristics within Schizaeaceae. Together with undescribed permineralized fossils recently discovered in Japan and southern South America, *Escuderia* provides a starting point for reconstructing the holophylogeny (Nishida *et al.*, 2024) of the group combining detailed anatomical evidence and molecular data.

The silicified palaeosol matrix containing *Escuderia* preserves a rich assemblage of both autochthonous and parautochthonous permineralized plant remains. These include a female reproductive organ of a gymnosperm tentatively comparable to Ginkgoales, shoots and leaves assignable to Araucariales (Araucariaceae and Podocarpaceae), and root systems with associated mycorrhizae. These findings not only add new elements to the flora previously described from the fossil-bearing Williams Point Beds but also underscore the potential of similar permineralized matrices across Antarctica as valuable targets for future palaeobotanical research.

The finding strengthens the hypothesis that Antarctica may have played a significant role in the diversification of the leptosporangiate ferns as well as of other vascular plants in the period of rapid worldwide angiosperm diversification.

## Supplementary Material

mcaf261_Supplementary_Data

## Data Availability

Supplementary video data are available either in the article supplementary material or in the online version of the article at the publisher’s website.

## AI Assistance

Artificial intelligence was used as a translation aid in a limited part of this paper (i.e. abstract), without any writing help.
